# Effects of a metabolic optimized fast track concept (MOFA) on bowel function and recovery after surgery in patients undergoing elective colon or liver resection: a randomized controlled trial

**DOI:** 10.1186/s12871-019-0823-6

**Published:** 2019-08-17

**Authors:** Christopher Uhlig, Thomas Rössel, Axel Denz, Sven Seifert, Thea Koch, Axel Rüdiger Heller

**Affiliations:** 1 Department of Anaesthesiology and Critical Care Medicine, University Hospital Carl Gustav Carus at the Technische Universität Dresden, Fetscherstr. 74, 01307 Dresden, Germany; 2 Department of Gastrointestinal, Thoracic and Vascular Surgery, University Hospital Carl Gustav Carus at the Technische Universität Dresden, Dresden, Germany; 3 Department of General Surgery, University Hospital of Friedrich-Alexander-University, Erlangen, Germany; 4 Department of Thorax, Vascular and Endovascular Surgery, Chemnitz Hospital, Chemnitz, Germany; 5 Department of Anaesthesiology and Surgical Intensive Care Medicine, University Hospital Augsburg, Augsburg, Germany

**Keywords:** Omegaven, Abdominal surgery, Liver surgery, Colon surgery, Omega-3 fatty acids, ERAS, Recovery after surgery, Randomized controlled trial

## Abstract

**Background:**

Enhanced recovery after surgery programs (ERAS) using thoracic epidural anesthesia and perioperative patient conditioning with omega-3 fatty acids (n3FA), glucose control (GC) and on-demand fluid therapy, respectively, showed beneficial effects. In the MOFA- study these components were used together in patients undergoing colon or liver surgery. We hypothesized that the use of a perioperative MOFA program improves intestine function represented as time to the first postoperative bowel movement in adult patients compared to standard ERAS.

**Methods:**

After BfArM and IRB approval 100 patients were enrolled in this prospective randomized controlled trial. All patients received ERAS therapy (control). In addition, the MOFA group received 0.2 g/kg fish oil (Omegaven®), preoperatively, followed by a 48 h continuous infusion of 0.2 g/kg/d n3FA; and GC was kept below < 8 mmol/L. Pre- and postoperatively energy drinks were administered.

**Results:**

As compared to control group the MOFA concept resulted in an earlier onset of flatulence by 14 h (46.6 ± 25.7, 32.0 ± 17.9, *p* = 0.030, hours, control vs. MOFA, respectively). Effects on onset of bowel movement were not observed (74.5 ± 30.4, 66.4 ± 29.2, *p* = 0.163, hours, control vs. MOFA, respectively). The disease severity (SAPS II score; *p* = 0.720) as well as deployment of resources (TISS 28 score, *p* = 0.709) did not differ between groups. No statistic significant difference between MOFA and control group regarding inflammation, impairment of coagulation, length of hospital stay or incidence of postoperative surgical complications were observed.

**Conclusions:**

The MOFA concept did not result in an improvement of intestine function or faster recovery after elective colon or liver surgery compared to standard ERAS therapy. Omega-3 fatty acids showed no impairment of coagulation or improved resolution of inflammation. Further trials in a larger patient collective are needed to investigate potential beneficial effects of omega-3 fatty acids in abdominal surgery.

**Trial registration:**

This trial was prospectively registered at the European Union Clinical Trials Register (EuDraCT 2005–004814-33, date: 10-05-2005, https://www.clinicaltrialsregister.eu/ctr-search/search?query=2005-004814-33+).

**Electronic supplementary material:**

The online version of this article (10.1186/s12871-019-0823-6) contains supplementary material, which is available to authorized users.

## Background

Within the past decade enhanced recovery after surgery (ERAS) programs were established covering a multitude of surgical interventions. Besides others, a meta-analysis on ERAS in different type of hepatectomy clearly showed improved patient outcome [1]. Aside minimizing surgical stress and improving pain control one key success factor for further developments of ERAS programs is the increasing control of noxious circumstances such as overwhelming metabolism [2].

Overshooting early up-regulation of host defense after complex surgery induces severe tissue injury, potentially culminating systemic inflammatory response with unfavorable outcome [3, 4]. Omega-3 fatty acids (n3FA) are capable of dampening early hyperinflammatory processes, by changing cell to cell signal transduction as shown [5]. Later features of host defense, however, are enhanced by n3FA without inducing hyperinflammatory states.

The n3FA have shown their efficacy in the treatment of chronic and acute inflammatory diseases [3] due to their pleiotropic effects on inflammatory cell signalling pathways [6–8]. In a variety of experimental [9, 10] and clinical studies omega-3 fatty acids attenuated hyperinflammatory conditions and induced faster recovery [11]. By reducing length of antibiotic therapy and hospital stay [12], even in critically ill patients, net cost savings are possible from the use of n3FA despite higher expenditure for the prescriptions [13].

In a prospective, open label, multicenter case series in 661 patients receiving parenteral fish oil our group evaluated survival, length of intensive care unit (ICU) stay, hospital stay and use of antibiotics, with respect to the primary diagnosis and extent of organ dysfunction [12]. Compared to the subgroup receiving less than 0.05 g/kg/d of fish oil, significantly more patients survived when 0.1–0.2 g/kg/d were administered.

Based on this data, the present study was designed to evaluate the effectiveness of an ERAS program supplemented with a priming dose of fish oil in the preoperative evening and a 48 h continuous infusion of 0.2 g/kg/d (so called ‘Metabolic Optimized FAst track concept’ [MOFA]) starting at the time of inducing anesthesia in patients undergoing elective hepatic and colon surgery.

We hypothesized that the use of a perioperative MOFA program improves intestine function represented as time to the first postoperative bowel movement in adult undergoing elective colon or hepatic surgery compared to controls without MOFA. Second, we hypothesized that the use of a perioperative MOFA program results in faster postoperative recovery represented by lower Simplified Acute Physiology Score (SAPS II) [14] as well as Simplified Therapeutic Scoring System values (TISS 28) [15] in adult patients undergoing elective colon or hepatic surgery compared to controls without MOFA.

## Methods

The present prospective, single blind, mono-center, randomized controlled trial was approved by the competent authorities, Bundesinstitut für Arzneimittelsicherheit und Medizinprodukte (BfArM, GZ 61–3910-4,031,476), and Institutional Review Board of the Technische Universität Dresden (AZ 193112005), as well as registered at European Union Drug Review Agency Clinical Trials (EuDraCT 2005–004814-33). The manuscript is reported according to the CONSORT guidelines [16].

### Patients and randomization

Adult patients (18 < age > 85 years) undergoing elective colon (tumor or diverticulosis surgery) or liver surgery (liver tumors independent from dignity without biliodigestive anastomosis) in the Universtiy Hospital Carl Gustav Carus (at the Technische Universität Dresden, Dresden, Germany) were eligible for the trial. Exclusion criteria were summarized in Additional file 1: Table S1. Convenience sampling was used as recruiting strategy and subjects were included over a three year period. Patients were randomized in blocks of four generated by a computer program either to standard ERAS (control) or MOFA group. To achieve homogeneity between groups an a priori stratification of the randomization was performed according to three strata: surgical procedure (colon or liver), body mass index (BMI, < 30 or ≥ 30) and perioperative risk classified as low or high, if one of the following factors was present: a previous abdominal operation, American Society of Anesthesiology Physical Status 3 or higher, central liver surgery, liver tumor > 5 cm. Group allocation was concealed in sequentially numbered opaque closed envelopes.

### Experimental protocol

**Fig. 1:**
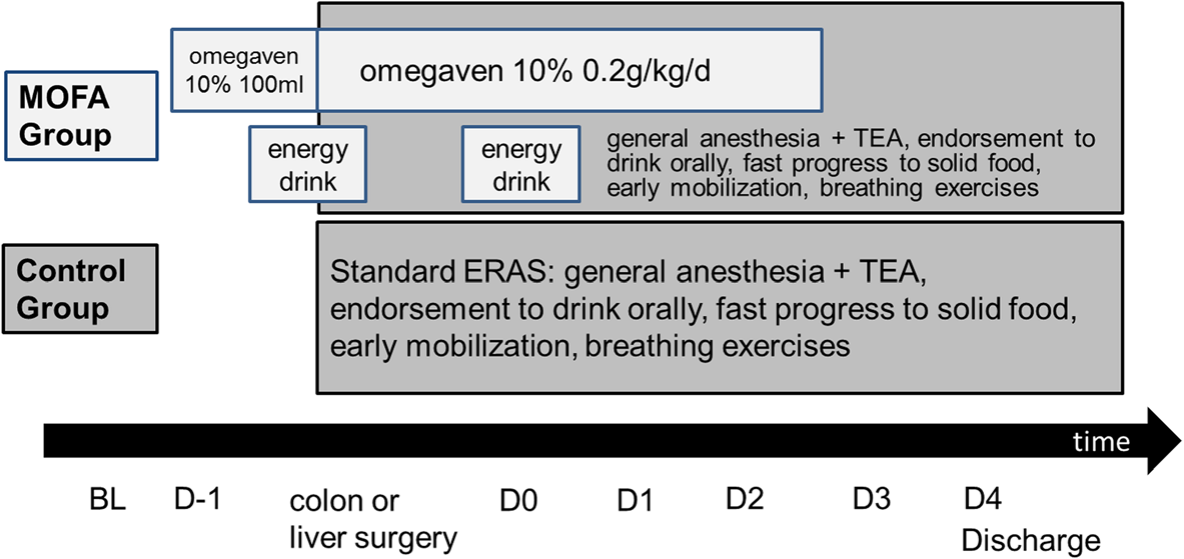
Time course of interventions After enrollment in the trial baseline values were taken and patients were randomized to control or MOFA group. All patients received a standard ERAS protocol with enhanced physiotherapy, short or no postoperative intensive care unit stay and planned discharge on the forth postoperative day. In addition, the MOFA group received a priming dose of 100 ml omegaven 10% on the evening before surgery. Two hours before the induction of anesthesia continuous infusion of omegaven 10% with 0.083 ml/kg/h (0.2 g/kg/d) for 48 h was started and a high caloric energy drink administered orally in the MOFA group. ERAS: enhanced recovery after surgery concept, MOFA: metabolic optimized fast track concept, TEA: thoracic epidural analgesia, BL: baseline, D-1: preoperative day, D0: day of surgery, D1-D4: postoperative day 1–4, respectively

Detailed interventions are described in Additional file 1: Table S2. A time course of intervention is depicted in Fig. 1.

### Preoperative care, anesthetic regimen and surgery

After enrollment in the trial baseline values were taken and patients were randomized to control or MOFA group. All patients received a standard ERAS protocol with enhanced physiotherapy, short or no postoperative ICU stay and planned discharge on the forth postoperative day. In addition, the MOFA group received a priming dose of 100 ml Omegaven® 10% (Fresenius Kabi AG, Bad Homburg, Germany) on the evening before surgery. Two hours before the induction of anesthesia continuous infusion of Omegaven® 10% with 0.083 ml/kg/h (0.2 g/kg/d) for 48 h was started and a high caloric energy drink (ProvideXtra® Drink 200 ml, Fresenius Kabi AG, Bad Homburg, Germany) administered orally in the MOFA group. All patients received general anesthesia with propofol/sufentanil for induction and volatile anesthetics in combination with sufentanil and thoracic epidural anesthesia (TEA) for maintenance. The choice of the volatile anesthetic and the neuromuscular blocking agent for continuous muscle relaxation was on the discretion of the anesthesiologist. Co-priming with 500 ml colloid solution before the first injection of local anesthetic for the epidural anesthesia was performed in the MOFA group. An arterial line as well as a central venous catheter were placed in all patients. Intraoperative fluid was restricted to 4 ml/kg/h. Surgery was carried out by a predefined experienced team of specialists in abdominal surgery. All patients who underwent liver surgery received a transverse upper abdomen laparotomy and in case of colon surgery a median laparotomy or laparoscopic approach as appropriate. Patients were extubated directly after the end of surgery. Intraoperative blood glucose level was kept below 8 mmol/L.

### Postoperative management

Patients who received colon surgery and basic liver surgery were transferred to the post anesthesia care unit and then to normal ward. Subjects undergoing more complex liver surgery were allocated to the ICU directly after end of anesthesia. On the day of surgery oral fluids were administered postoperatively from 2 h on. In the MOFA group the high caloric energy drink was given and Omegaven® 10% infusion was stopped at 2nd postoperative day. Patients were endorsed to drink > 1500 ml per day. A fast progress to solid food was intended. Physical mobilization and breathing exercises started 5 h after the end of surgery and were continued until discharge. Infusion was limited to 500 ml per day. TEA was continued as patient controlled analgesia until the 2nd postoperative day and removed together with the central venous line. Blood sugar level was aimed to be kept between 4 mmol/L and 8 mmol/L in the MOFA group and between 4 mmol/L and 10 mmol/L in the control group during the first three postoperative days (Additional file 1: Table S3). Discharge was aimed from the fourth postoperative day on.

### Safety thresholds

Participation in the trial was terminated at any time point, if one of the following dropout criteria was present: request of the patient, therapy refractory bowel paralysis, allergic reaction to study medication, onset of contraindications against enteral nutrition, development of septic shock and violation of safety parameters. Safety parameters to avoid bleeding complications were measured daily during the first three postoperative days. The following safety thresholds were defined: hemoglobin > 4.5 mmol/L, platelets > 50 Gpt/L, prothrombin time (PT) > 50%, activated partial thromboplastin time (aPTT) < 60 s. If a safety threshold was violated, Omegaven® infusion was stopped and reasons for violation were investigated.

### Measurements and data collection

Daily visits throughout the study period were performed. Investigators collecting data were not blinded to groups. Patients were unaware of group allocation but blinding could be easily broken. Preoperative baseline data included physical examination, basic monitoring (blood pressure, peripheral oxygen saturation) and laboratory parameters (complete blood count, aPPT, PT, antithrombin, fibrinogen, blood urea nitrogen, creatinine, c-reactive protein (CRP), procalcitonin (PCT), tumor necrosis factor-alpha (TNF-α), interleukin 6 (IL-6) and 10 (IL-10), blood sugar, lactate). These measurements were repeated until the third postoperative day. Data from the medical record were extracted daily. The presence of flatulence and/or bowel movement was documented during the first postoperative days. The TISS 28 and SAPS II scores were calculated daily.

### Statistics

All calculations were performed using SPSS (IBM SPSS Statistics Vers. 23.0.0.0, IBM Deutschland GmbH, Ehningen, Germany). Graphs were computed with GraphPad Prism (Vers. 6.01, GraphPad Software Inc., La Jolla, CA, USA). Sample size was calculated based on an internal database consisting of 291 patients undergoing colon surgery, where the time to the first bowel movement was 3.25 ± 1.7 days. With an assumption of a two-sided α of 0.05 and a statistical power of 0.80, 47 patients per group were calculated to be necessary to accelerate the onset of bowel movement (primary endpoint) by one day. To compensate for dropouts, a sample size of 50 per group was projected. Distribution of the data was assessed visually using Q-Q-Plot. Patient data were analyzed as intention to treat. No assumption for missing data was done. Values are given as median, 25% percentile, 75% percentile, minimum and maximum; mean ± standard deviation or absolute number (percentage); as appropriate. The analysis of the primary endpoint was performed with log rank test and cox regression. A two-sided Student’s t-test or Mann-Whitney U test were used as appropriate for binary outcomes. Statistical significance between groups for repeated measures as well as time effect and time vs. group effect were calculated with a general linear model using the respective baseline data as covariate. Statistical significance was considered to be at two-sided *p* < 0.05.

## Results

**Fig. 2:**
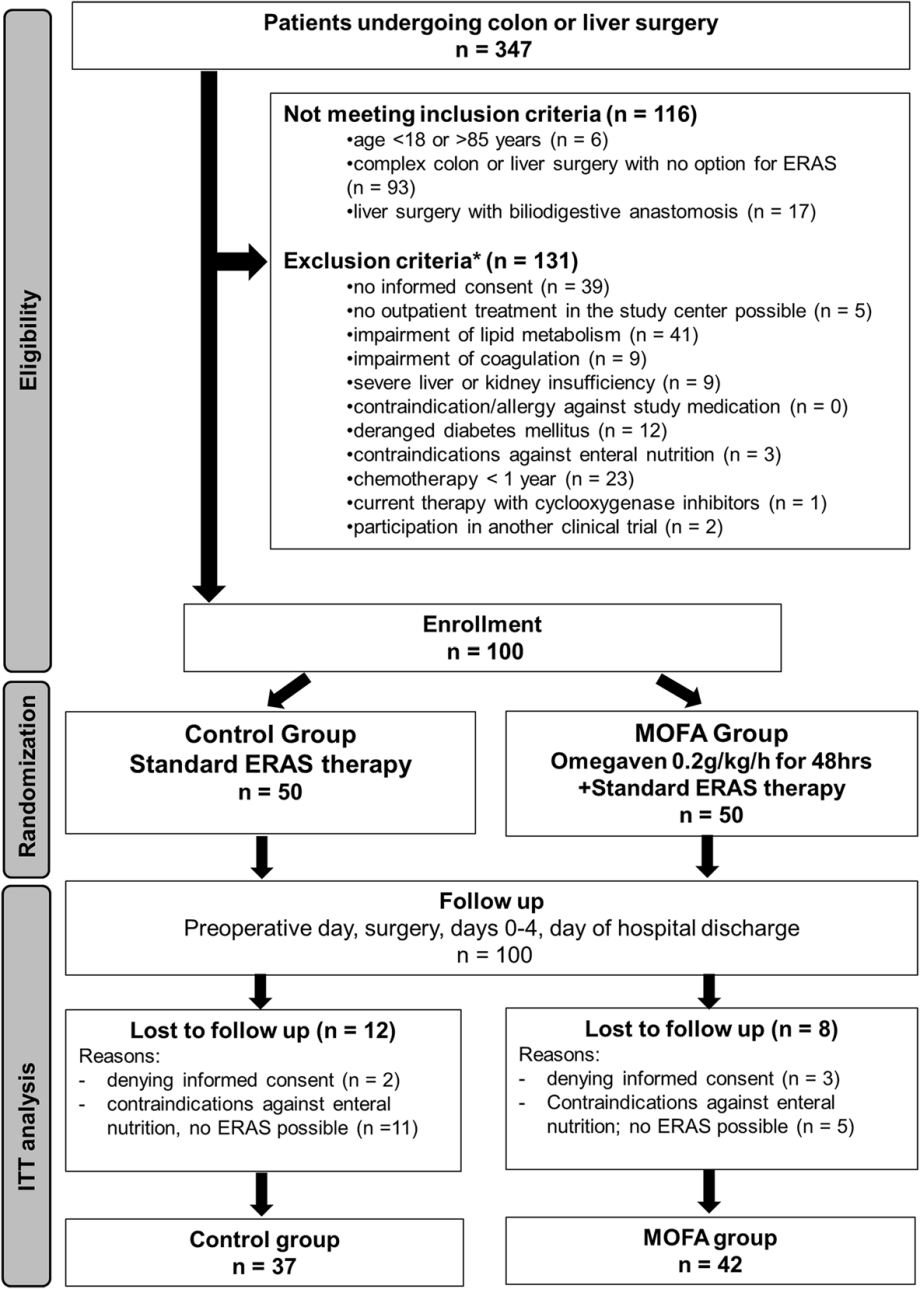
Flowchart. Consecutive patients undergoing open liver or colon surgery were enrolled in the trial and randomized into ERAS and MOFA group. During the follow up period 28 patients were lost. Finally, 79 patients were analyzed in the per protocol analysis. ERAS: enhanced recovery after surgery concept, MOFA: metabolic optimized fast track concept, ITT: intention to treat

The consort diagram is depicted in Fig. 2. Out of 347 screened patients, 100 consecutive patients were enrolled in the trial. Various dropouts, mainly caused by more complex resections than planned due to intraoperative tumor spread, resulted in 79 patients (*n* = 37 control, *n* = 42 MOFA group) with available follow up data. Baseline characteristics are summarized in Table 1.

**Table 1:** Baseline characteristics

	Control group (*n* = 50)	MOFA group (n = 50)
Age [years]	61 ± 12	60 ± 19
Gender [no./%]		
Female	16 (32.0)	18 (36.0)
Male	34 (68.0)	32 (64.0)
Body height [m]	1.73 ± 0.09	1.70 ± 0.09
Body weight [kg]	82.7 ± 16.2	80.5 ± 15.7
BMI [kg/m^2^]	27.6 ± 4.7	27.9 ± 4.2
Surgical procedure [no./%]		
Colon	30 (60.0)	30 (60.0)
Liver	20 (40.0)	20 (40.0)
Surgical technique [no./%]		
Laparoscopic	6 (12.0)	5 (10.0)
Open	43 (86.0)	45 (90.0)
Conversion^a^	1 (2.0)	0 (0.0)
ASA [no./%]		
I	2 (4.0)	8 (16.0)
II	29 (58.0)	30 (60.0)
III	19 (38.0)	12 (24.0)
Preconditions		
IDDM	2 (4.0)	3 (6.0)
NIDDM	6 (12.0)	4 (8.0)
Arterial hypertension	29 (58.0)	25 (50.0)
Coronary heart disease	2 (4.0)	6 (12.0)
COPD	1 (2.0)	0 (0.0)

Values are given as absolute number (percentage) or mean ± standard deviation, as appropriate. *ASA* American Society of Anesthesiology physical status, *BMI* Body mass index, *COPD* Chronic obstructive pulmonary disease, *IDDM* Insulin dependent diabetes mellitus, *MOFA* Metabolic optimized fast track concept, *NIDDM* Non-insulin dependent diabetes mellitus, ^a^: intraoperative conversion from laparoscopic to open abdominal surgery

### Bowel function

Postoperative bowel function is shown in Table 2. With the MOFA concept an earlier onset of discharge of gas by 14 h (46.6 ± 25.7, 32.0 ± 17.9, *p* = 0.030, hours, control vs. MOFA, respectively) was detected. However, there was no statistic significant difference in bowel movement (74.5 ± 30.4, 66.4 ± 29.2, *p* = 0.163, hours, control vs. MOFA, respectively) or the administration of agents for bowel stimulation.

**Table 2:** Postoperative intestinal function

	Control group	MOFA group	*P* value
First bowel movement [h]	74.5 ± 30.4 [38]	66.4 ± 29.2 [43]	0.163
First discharge of gas [h]	46.6 ± 25.7 [36]	32.1 ± 17.9 [43]	0.030
Bowel sounds [h]	40.8 ± 14.6 [40]	34.4 ± 14.1 [44]	0.042
Onset of enteral nutrition [days]	0.0 (0.0, 0.0, 1.0, 5.0) [42]	0.0 (0.0, 0.0, 1.0, 3.0) [43]	0.252
Bowel stimulation^a^ [days of onset]	2.0 (0.0, 0.0, 3.0, 6.0) [39]	2.0 (0.0, 0.0, 3.0, 5.0) [43]	0.703
Bowel stimulation^a^ [n]	20 (51.3) [39]	22 (51.2) [43]	1.000

Values are given as mean ± standard deviation, median (minimum, 25% percentile, 75% percentile, maximum) or absolute number (percentage) as appropriate. Number of analyzed patients can be found in brackets. Statistical significance was considered to be at two-sided *p* < 0.05. Differences between groups were tested using log rank test and cox regression. Frequencies were analyzed using Fisher’s Exact test or Chi square test, as appropriate. *MOFA* Metabolic optimized fast track concept, ^a^: bowel stimulation includes pharmacological and osmotic/mechanical stimulation

### Recovery after surgery

**Fig. 3:**
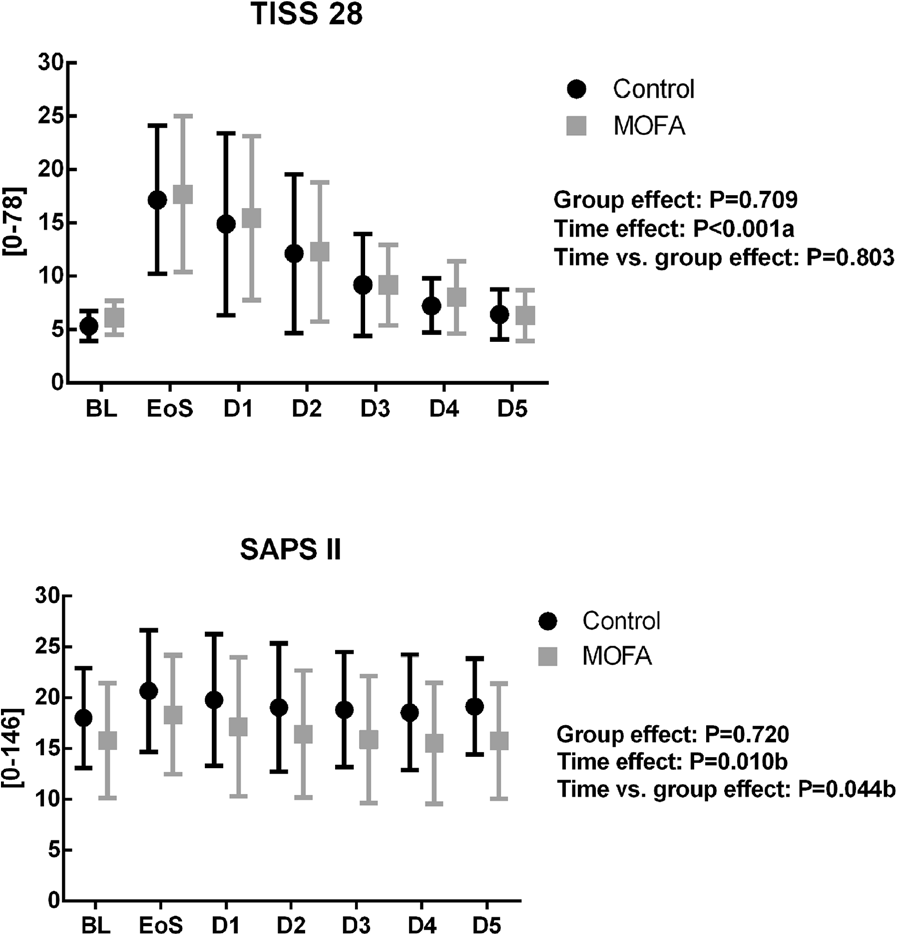
TISS 28 and SAPS II score. Values are given as mean ± standard deviation. Statistical significance was considered to be at two-sided *p* < 0.05. Differences between groups, as well as time and time vs. group effect were tested using a general linear model with the respective baseline value as covariate. a: linear effect, b: quadratic effect, BL: baseline, EoS: Evening of surgery, D1-D5:postoperative day 1–5, respectively. SAPS: Simplified Acute Physiology Score, TISS: Simplified Therapeutic Scoring System, MOFA: metabolic optimized fast track concept

As shown in Fig. 3, no statistic significant difference in disease severity, represented by the SAPS II score was observed between control and MOFA group (*p* = 0.720). The deployment of resources measured by the TISS 28 score did not differ between groups (Fig. 3). Duration of anesthesia and surgery, intraoperative blood loss, overall amount of administered crystalloids and transfusions were comparable between groups (Table 3). Patients undergoing liver surgery received more crystalloids in the control group compared to MOFA group. Postoperative surgical complications were higher in the control group causing trial dropouts mainly due to insufficiency of anastomosis with consecutive reoperation and impossible enteral nutrition (Table 3). There were no statistic significant differences, neither in length of ICU nor in length of hospital stay (Table 3). There was one in-hospital death in the control group due to myocardial infarction with consecutive multi-organ failure after cardiac arrest.

**Table 3:** Duration of Surgery, Anesthesia, ICU and hospital stay, Complications of surgery

	Control group	MOFA group	*P* value
Duration of surgery			
[min]			
All	190 (92,157, 265, 529) [50]	215 (89, 158, 310, 560) [50]	0.425
Colon	175 (92, 150, 203, 293) [30]	206 (89, 153, 268, 482) [30]	0.240
Liver	235 (130, 169, 397, 528) [20]	221 (92, 166, 336, 560) [20]	0.654
Duration of anesthesia			
[min]			
All	276 (180, 239, 355, 620) [50]	300 (176, 245, 418,632) [50]	0.491
Colon	262 (180, 238, 309, 390) [30]	289 (187, 243, 361, 570) [30]	0.578
Liver	335 (196, 237, 537, 620) [20]	323 (176, 245, 465, 632) [20]	0.186
Intraoperative blood loss			
[ml]			
All	175 (0, 30, 1025, 3000) [50]	400 (0, 30, 950, 3000) [49]	0.775
Colon	50 (0, 30, 200, 1150) [30]	30 (0, 20, 100, 3000) [29]	0.399
Liver	1100 (100, 613, 1438, 3000) [20]	950 (50, 525, 1950, 2820) [20]	0.909
Need for transfusion^b^ [no]			
All	10 (20.0) [50]	6 (12.0) [50]	0.414
Colon	2 (6.7) [30]	1 (3.3) [30]	1.000
Liver	8 (40.0) [20]	5 (25.0) [20]	0.501
Transfusion products			
PRBC [units]	4.0 (2.0, 2.5, 5.0, 8.0) [9]	2.5 (1.0, 2.0, 4.0, 6.0) [8]	0.288
FFP [units]	4.0 (2.0, 2.0, 5.25, 9.0) [6]	3.0 (2.0,2.0, 4.0, 4.0) [4]	n.a.
Cumulative fluid therapy			
Crystalloids [ml]			
All	2500 (500, 1375, 3500, 5900) [50]	1750 (0, 1100, 2500, 6500) [49]	0.054
Colon	1500 (500, 1000, 2500, 5500) [30]	1500 (0, 1000, 2250, 6500) [29]	0.758
Liver	3500 (2000, 3000, 4000, 5900) [20]	2263 (0, 1500, 3500, 5300) [20]	0.004
Colloids [ml]			
All	500 (0, 500, 1500, 2500) [50]	1000 (0, 500, 1500, 3000) [49]	0.043
Colon	500 (0, 0, 1000, 2000) [30]	500 (0, 500, 1000, 3000) [29]	0.005
Liver	1500 (500, 500, 1500, 2500) [20]	1500 (0, 500, 2000, 3000) [20]	0.504
Hospital length of stay [days]			
All	8.0 (6.0, 8.0, 9.0, 20.0) [37]	8.0 (6.0, 8.0, 9.0, 20.0) [43]	0.466
Colon	8.0 (6.0, 7.0, 10.0, 30.0) [23]	8.0 (6.0, 7.0, 9.0, 12.0) [27]	0.651
Liver	8.0 (6.0, 8.0, 9.0, 20.0) [14]	9.0 (7.0, 8.8, 10.3, 13.0) [16]	0.860
ICU length of stay [hours]			
All	34 (21, 23, 49, 67) [14]	27 (12, 20, 45, 80) [15]	0.237
Colon	49 [1]	30 (12, 13, 43, 44) [4]	n.a.
Liver	25 (21, 22, 49, 67) [13]	27 (18, 21, 45, 88) [11]	0.558
Postoperative surgical complications^a^ [no.]			
Bleeding	0 (0.0) [50]	1 (2.0) [50]	1.000
Insufficiency of	4 (8.0) [50]	0 (0.0) [50]	0.118
anastomosis			
Wound infection	3 (6.0) [50]	0 (0.0) [50]	0.242
Non-infective wound	1 (2.0) [50]	1 (2.0) [50]	1.000
complication			
Biliary fistula	0 (0.0) [50]	0 (0.0) [50]	n.a.
Reoperation	4 (8.0) [50]	0 (0.0) [50]	0.118
other	2 (4.0) [50]	2 (4.0) [50]	1.000
In-hospital death [no.]	1 (2.0) [50]	0 (0.0) [50]	1.000

Values are given as mean ± standard deviation, median (minimum, 25% percentile, 75% percentile, maximum) or absolute number (percentage) as appropriate. Number of analyzed patients can be found in brackets. Statistical significance was considered to be at two-sided *p* < 0.05. Differences between groups were tested using a Student’s *t*-test or Mann-Whitney *U* test as appropriate. Frequencies were analyzed using Chi-square or Fisher’s Exact test, as appropriate. *FFP* Fresh frozen plasma, *ICU* Intensive care unit, *MOFA* Metabolic optimized fast track concept, *PRBC* packed red blood cells. ^a^: Postoperative surgical complications are provided during whole hospital stay or until dropout. ^b^: need for transfusion is given during whole hospital stay or until dropout

### Hemodynamics, clinical chemistry and metabolic data

Hemodynamics are summarized in Additional file 1: Table S4. No difference between groups were detected. The blood sugar level was kept below 10 mmol/L in the control group and below 8 mmol/L in the MOFA group (Additional file 1: Figure S1) resulting in a higher insulin administration in the MOFA group (Additional file 1: Table S5). There was no difference in serum values of parameters of liver injury and function (Additional file 1: Figure S3). Both groups had comparable kidney function (Additional file 1: Table S6).

### Inflammation

Pro- and anti-inflammatory cytokines as well as clinical relevant parameters of inflammation are depicted in Additional file 1: Figure S4. No difference between groups in CRP, PCT, IL-6, TNF-α or IL-10 were observed in the MOFA group compared to control.

### Safety thresholds and investigational drug related adverse events

Coagulation parameters were comparable between groups (Additional file 1: Figure S2) and intraoperative blood loss was comparable. Severe postoperative bleeding complications did not occur in the MOFA group compared to control measured on need for transfusion (Table 3) and hemoglobin and hematocrit levels were comparable between groups (Additional file 1: Table S6). However, in one patient, who underwent liver surgery with unplanned high intraoperative blood loss, Omegaven® was stopped precautiously on the first postoperative day by the treating intensivist although safety thresholds were not violated.

## Discussion

The current trial is an independent investigator-initiated trial focusing on perioperative effects of the MOFA program. The trial was controlled and the interventions were simple and feasible, which improves external validity. The research question is highly relevant considering around 2.1 million patients treated for gastrointestinal diseases in Germany every year (according to hospital database of the German Federal Agency for Statistics – Statistisches Bundesamt) resulting in approximately 8500 EUR up to 11,800 EUR costs per colon procedure and approximately 10,000 EUR up to 15,500 EUR costs per liver procedure [17–19].

### Major findings

Major findings of the present trial are

1. The MOFA concept did not result in an improvement of intestine function represented as time to the first postoperative bowel movement in adult patients undergoing elective colon or hepatic surgery compared to standard ERAS therapy.
2. A faster recovery after surgery measured by the SAPS II score was not observed in the MOFA group compared to control group.
3. Omegaven® did not cause statistic significant impairment of coagulation or higher intraoperative blood loss compared to standard ERAS therapy.

### ERAS programs in abdominal surgery

Careful analgesia, early enteral feeding and early mobilization are one of the key stones of modern fast track surgery [20]. The ERAS programs in abdominal surgery improve patient outcome and reduces costs [21, 22]. However, the comparability of the different ERAS approaches were questioned [22]. In the present trial our standard ERAS program consists of various interventions all recommended by the ERAS Society [20]. One essential part of our standard ERAS program is epidural anesthesia. Thoracic epidural anesthesia enhances gastrointestinal transit and reduces postoperative pain compared to conventional opioid analgesia [23]. The placement of epidural catheters in patients undergoing laparoscopic surgery might be obsolete considering risks and benefits as well as the alternative treatment with systemic opioids and/or local wound infiltration [24]. Furthermore, epidural anesthesia may be contraindicated in case of anticoagulant therapy [25], whereby various patients may be excluded from our proposed standard ERAS or MOFA program in case of early begin of anticoagulant therapy especially after laparoscopic surgery. In addition, new regional anesthetic techniques like the transverse abdominis plane block (TAP) or the paravertebral block, which are less invasive and have in case of TAP block less contraindications regarding anticoagulation showed comparable or better results with respect to analgesic effect or complications compared to TEA [26, 27]. However, a careful analgesia without influencing gastrointestinal transit may be a key concept independent of the type of analgesia. In addition, perioperative blood glucose control is essential. Kotagal and colleagues found in a retrospective cohort study in more than 40,000 patients undergoing abdominal surgery that perioperative hyperglycemia is associated with more adverse events even in patients without diabetes mellitus [28]. Therefore, the MOFA group received more insulin than the control group, but without resulting in lower blood glucose levels compared to control group. However, the average blood sugar level was below 8 mmol/L for most of the measurement points in both groups.

### Influence of omega-3 fatty acids

Host defense is one of the most complexly regulated systems within the mammalian organism. Multitudes of back coupling mechanisms are responsible for up-regulating the immune response and for subsequent shut down of hostile responses during recovery. Through several positive feedback loops early host defense can induce hyperinflammatory states. Counterbalancing antagonistic systems are induced with a time delay. The early phase (0–72 h) is characterized by the predominance of pro-inflammatory eicosanoids, cytokines (e.g. TNF-α, IL-1, IL-6, IL-8) and the in parts overlapping later phase by the predominance of anti-inflammatory cytokines (IL-4, IL-10, IL-13 and transforming growth factor-β) [29, 30]. This time course of cytokine production can differ in patients with recurrent septic episodes.

The addition of Omegaven® to the standard ERAS treatment was promising due to several of beneficial immunomodulatory effects described in the literature. Omegaven® is a lipid parenteral formula which contains 10 g fish oil/100 ml which correspondingly has 1.25–2.82 g eicosapentaenoic acid (EPA, 20:5 n-3) and 1.44–3.09 g docosahexaenoic acid (DHA, 22:6 n-3) [31]. The n-6:n-3 PUFA ratio in Omegaven® is 1:6–8.

In the recent years a couple of meta analyses in the field of intravenous n3FA administration shed light on their efficacy in surgical patients [32–34] as well as in critical illness [32, 35, 36]. Significantly lower infection rates, ICU stay, and length of hospital stay have concordantly been identified in three recent meta-analyses in surgical patients [32, 37, 38]. In this regard the post-traumatic metabolism after gastrointestinal cancer surgery was improved [4], which may in part be explained by resolvin E_1_ activity [39]. This complex regulation is conferred by reduced release of pro-inflammatory arachidonic acid-derivatives and platlet-activating factor, and, on the other hand, by the amplification of anti-inflammatory EPA-derivatives, which lower the formation of cytokines such as TNF-α and IL-1 [40], without inhibiting phagocytosis, burst activity, or bactericidal activity [41]. The observed modulation of inflammation by n3FA, thus, cannot unequivocally be assigned to the pro- or anti-inflammatory side. While early pro- inflammatory eicosanoids are down- regulated, later on, cellular host defense mechanisms are augmented.

Besides the physiological rationale and experience from small clinical trials, Omegaven® failed to improve postoperative intestinal function, inflammation and recovery from surgery in the present trial. We noticed neither an aggravation nor depression of immune function represented by cytokine levels and clinical relevant inflammatory parameters. In our opinion, four different reasons could explain those findings: 1) the trial was underpowered; 2) the dosage of Omegaven® was too low; 3) the start of Omegaven® was too late or the duration of administration was too short and 4) there is no clinical favourable effect of Omegaven® regarding intestinal function, inflammation or recovery after surgery.

Several factors may contribute to low power in the present trial. The estimated compensation of 6% for dropouts by three subjects per group was too low. Both the ERAS and MOFA program consists of various interventions, which may lead to patient inconvenience and therefore denying consent during the trial. In addition, the time of first bowel movement after colon surgery, which was used for sample size calculation may be different between from those after receiving liver surgery. However, since a statistic significant difference in the onset of flatulence was observed in the MOFA group, the authors consider the statistical power as main reason for failing the primary endpoint.

Finding the correct dosage of study medication in clinical trials is challenging. Heller et al. conducted a large observational study of intravenous fish oils in 661 patients from 82 German hospitals [12]. Doses between 0.05–0.2 g/kg/day of fish oil were given to mixed hospital patients for at least 3 days. An average of 0.11 g/kg/day [range 0.005–0.426 g/kg/day] was administered for an average of 8.7 ± 7.5 days. Fish oil administration significantly lowered hospital mortality from 18.9 to 12% as predicted by SAPS II scores. Fish oil doses > 0.05 g/kg/day significantly decreased length of ICU and hospital stay. Doses between 0.1–0.2 g/kg/day was associated with significantly higher survival rates compared to doses < 0.05 g/kg/day. Finally, a lowered antibiotic demand of 26% was observed in patients receiving 0.15–0.2 g/kg/day of fish oil. Based on this trial we opted for Omegaven® dosage. Wang et al. administered up to 10 g/day by giving 0.15–0.2 g/kg/day of fish oil from Omegaven® in 40 adult patients with severe acute pancreatitis [42]. Clinical parameters were measured on day 6, the following day after the intervention. In the fish oil treated group, EPA was significantly higher in plasma membranes, CRP concentrations were lower and oxygenation index were higher compared to control. However, an optimal dose finding trial should be performed before conducting future clinical trials involving intravenous Omegaven®.

The optimal time point for beginning, the duration as well as the route of administration of n3FA is still crucial. As shown by Brower et al., the intestinal resorption of n3FA is not sufficient in all patients during systemic inflammation [43]. Therefore, an intravenous administration is favorable. The optimal start and duration of supplementation with n3FA was chosen to cover the peak of inflammatory response. However, an orally supplementation in a longer period before surgery may enhance beneficial effects of n3FA. Furthermore, recent orally available n3FA formulas consisting of monoglycerids attached with EPA (EPA/DHA ratio 10:1) and less fishy taste may be opted for oral supplementation before and after surgery [44].

Recent randomized controlled trials showed no effect on mortality in critical ill or cancer patients undergoing elective surgery [45, 46]. These findings are supported by metaanalysis, which showed no benefit on mortality or infectious complications [36]. However, there is the possibility that n3FA do not have an effect on intestinal function, resolution of inflammation or recovery after surgery in the currently investigated patient collective. Nevertheless, in awareness that the present trial is underpowered a generalization of the results cannot be made.

### Safety of Omegaven®

The anti-thrombotic and anti-atherogenic effects of fish oil have raised potential safety concerns due to decreased blood viscosity, which could decrease blood clotting [47]. To date, negative clinical data is sparse for prolonged bleeding times for fish oil alone, or in combination with blood thinners [48–50] with the exception of some isolated case reports on possible interactions between warfarin and/or trazodone and/or aspirin with oral fish oil [51–54]. Fish oil supplementation has shown to be safe in various RCTs in conjunction with and without anti-coagulant therapy. In the GISSI multi-center trial, no negative blood thinning interactions were reported in over 10,000 post-myocardial patients that consumed around 1 g/day DHA + EPA from fish oil in combination with blood thinning agents for 3–5 years [55]. In high risk surgical patients undergoing major vascular surgeries, femoral oral artery puncture or percutaneous transluminar coronary angioplasty, Harris reported on 20 studies which included over 4300 subjects that supplemented between 1 and 21 g/day fish oil concomitantly with blood-thinning medications in subjects [48]. Of these, 5 bleeding complications were reported and no clinically relevant extended bleeding times were documented.

Statistical significant undesirable effects of n3FA on coagulation or bleeding disorders did not occur in the present trial. However, because the present trial is underpowered, further trials are needed to investigate the safety of Omegaven® regarding impairment of coagulation.

### Limitations

The present trial has several limitations. First, the trial was an open label trial, which may have and severe impact on outcome [56]. Blinding of outcome assessors and patients via a double dummy design with sham infusion of crystalloid infusion would have improved drastically trial costs for camouflaging study medication. Second, the trial is underpowered due to the high rate of dropouts in both groups as discussed before. Third, liver and colon surgery may be too heterogeneous since higher blood loss and transfusion are more likely to occur in liver surgery. We opted for a stratified randomization strategy, which enrolled an equal number of patients with liver resection in both groups which enhanced comparability and improved external validity of the trial. Forth, only a few patients received laparoscopic surgery in our center, whereby the use of laparoscopic techniques is improving for simple and also complex liver and colon surgery. In addition, less surgical trauma may be caused by laparoscopic techniques resulting in moderated inflammatory response confounding the study results. Fifth, the placement of epidural anesthesia in patients undergoing laparoscopic surgery might be obsolete considering risks and benefits as well as the alternative treatment with systemic opioids and/or local wound infiltration as mentioned before [24]. Furthermore, epidural anesthesia may be contraindicated in case of anticoagulant therapy [25], whereby various patients may be excluded from our proposed standard ERAS or MOFA program in case of early begin of anticoagulant therapy especially after laparoscopic surgery. However, considering a trial design without obligate TEA, an imbalance between groups with different number of TEA would be a clear confounder, due to enhanced bowel movement and shorter hospital stay by TEA after abdominal surgery [44]. Sixth, the evaluation of safety thresholds might be difficult in case of large blood loss caused by surgery.

### Implications for further studies

Further trials investigating the influence of n3FA in a larger patient collective undergoing abdominal surgery are warranted. Such a trial should be randomized controlled with a double dummy design, focusing on the effects of omega-3 fatty acids in patients undergoing open abdominal surgery. An optimal dose finding trial as well as the optimal time point for beginning of the Omegaven® treatment is necessary. A preoperative start of orally n3FA supplementation for instance two weeks before surgery and a longer supplementation period of for instance 4 weeks postoperatively may enhance the immunomodulatory effect of n3FA. An increase in daily dosage to 0.5 g/kg/d fish oil may result in a clinical relevant improvement of intestinal function and faster recovery after surgery.

## Conclusions

The metabolic optimized fast track concept did not result in an improvement of intestine function or faster recovery after elective colon or liver surgery compared to standard enhanced recovery after surgery program. Omega-3 fatty acids showed no impairment of coagulation or improved resolution of inflammation. Further trials in a larger patient collective are needed to investigate potential beneficial effects of omega-3 fatty acids in abdominal surgery.

## Additional file

Additional file 1: Supplemental digital content for this manuscript is provided. This file contains a detailed description of methods (**Table S1.** Exclusion criteria, **Table S2.** Detailed description of study intervention, **Table S3.** Blood glucose management) as well as additional results (**Figure S1.** Blood glucose levels, **Figure S2.** Coagulation parameters, **Figure S3**: Parameters of liver injury and function, **Figure S4.** Inflammation, **Table S4.** Hemodynamic data, **Table S5.** Insulin therapy, **Table S6.** Additional clinical chemistry and blood count). (DOCX 452 kb)

## Data Availability

The datasets used and/or analyzed during the current study are available from the corresponding author on reasonable request.

## Abbreviations

aPTT: Activated partial thromboplastin time
BfArM: Bundesinstitut für Arzneimittelsicherheit und Medizinprodukte
CONSORT: Consolidated Standards of Reporting Trials
CRP: C-reactive protein
DHA: Docosahexaenoic acid
EPA: Eicosapentaenoic acid
ERAS: Enhanced recovery after surgery
EuDraCT: European Union Drug Review Agency Clinical Trials
GC: Glucose control
ICU: Intensive care unit
IL: Interleukin
MOFA: Metabolic Optimized FAst track concept
n3FA: Omega-3 fatty acids
PCT: Procalcitonin
PT: Prothrombin time
RCT: Randomized controlled trial
SAPS: Simplified Acute Physiology Score
SOFA: Sequential/Sepsis related Organ Failure Assessment
TAP: Transverse abdominis plane block
TEA: Thoracic epidural anesthesia
TISS: Simplified Therapeutic Scoring System
TNF-α: Tumor necrosis factor-alpha
