# Supplementary material for: Effects of a metabolic optimized fast track concept (MOFA) on bowel function and recovery after surgery in patients undergoing elective colon or liver resection: a randomized controlled trial
Source: BMC Anesthesiol. 2019 Aug 17;19:156. doi: 10.1186/s12871-019-0823-6 (PMC6698338; doi:10.1186/s12871-019-0823-6)
Supplement: Supplementary file 1 — Supplemental digital content for this manuscript is provided. This file contains a detailed description of methods (Table S1. Exclusion criteria, Table S2. Detailed description of study intervention, Table S3. Blood glucose management) as well as additional results (Figure S1. Blood glucose levels, Figure S2. Coagulation parameters, Figure S3: Parameters of liver injury and function, Figure S4. Inflammation, Table S4. Hemodynamic data, Table S5. Insulin therapy, Table S6. Additional clinical chemistry and blood count). (DOCX 452 kb) [file 12871_2019_823_MOESM1_ESM.docx]

**Additional File 1**

**Effects of a Metabolic Optimized Fast Track Concept (MOFA) on bowel function and recovery after surgery in patients undergoing elective colon or liver resection: a randomized controlled trial.**

Christopher Uhlig^1^, Thomas Rössel^1^, Axel Denz^2,3^, Sven Seifert^2,4^, Thea Koch^1^, Axel R. Heller^1,5^

^1^Department of Anaesthesiology and Critical Care Medicine, University Hospital Carl Gustav Carus at the Technische Universität Dresden, Dresden, Germany; ^2^Department of Gastrointestinal, Thoracic and Vascular Surgery, University Hospital Carl Gustav Carus at the Technische Universität Dresden, Dresden, Germany; ^3^Department of General Surgery, University Hospital of Friedrich-Alexander-University, Erlangen, Germany; ^4^Department of Thorax, Vascular and Endovascular Surgery, Chemnitz Hospital, Chemnitz, Germany; ^5^Department of Anaesthesiology and Surgical Intensive Care Medicine, University Hospital Augsburg, Augsburg, Germany

*Corresponding author:*

Christopher Uhlig, MD, M.Sc, Department of Anaesthesiology and Critical Care Medicine, University Hospital Carl Gustav Carus at the Technische Universität Dresden, Fetscherstr. 74, 01307 Dresden, Germany Email: christopher.uhlig@ukdd.de, Tel: +49- 351 458-4145, Fax: +49- 821 458-4336.

**Table of Content**

**Detailed description of Material and Methods**

**Table S1 Exclusion criteria………………………………………………………………..………3**

**Table S2 Detailed description of study intervention………………………………...…….4-6**

**Table S3 Blood glucose management…………………………………………………………..7**

**Additional results**

**Figure S1 Blood glucose levels………………………………………………………………….9**

**Figure S2 Coagulation parameters………………………………………………….………….10**

**Figure S3 Parameters of liver injury and function………………………………….…….....11**

**Figure S4 Inflammation…………………………………………………………………….……..12**

**Table S4 Hemodynamic data..………………………………………………………….………..13**

**Table S5 Insulin therapy………………………………………………………………….……….14**

**Table S6 Additional clinical chemistry and blood count…………………………………...15**

Table S1: Exclusion Criteria

| **Exclusion Criteria** |
| --- |
| No informed consent, incompliance with the protocol or missing capacity to consent |
| No permanent residence in Dresden or no possibility to attend outpatient treatment in the recruiting center |
| Pregnancy or breast feeding |
| Contraindications against fish oil (allergy to fish protein) |
| Diseases of the fat metabolism |
| Bleeding disorders |
| Severe liver or renal insufficiency, defined as any type of acute liver failure or chronic liver disease such as liver cirrhosis stage B or C according to Child Pugh classification and serum creatinine level >177μmol/L. |
| Contraindications against hydroxylethyl starch (allergy against hydroxylethyl starch) |
| Contraindications against postoperative enteral feeding e.g. bleeding or obstruction distal of the feeding probe |
| Poorly regulated diabetes mellitus |
| Current cytostatic therapy or chemotherapy within the last year |
| Current therapy with cyclooxygenases inhibitors (≤ 5 days) |
| Consent to another interventional clinical trial |

Exclusion criteria were altered by an amendment to the study protocol before the first patient was enrolled. The following points were modified: The criteria “diseases of fat metabolism”, “severe liver or renal insufficiency” as well as “bleeding disorders” were added because low adherence to the ERAS protocol was expected in that patient population. LDL: low density lipoprotein, HDL: high density lipoprotein.

Table S2: Detailed description of the study intervention

| **Time point** | **Control Group**  **(Standard ERAS concept)** | **MOFA Group** |
| --- | --- | --- |
| Preoperative Management | - Diagnostics, Informed consent (surgical procedure, anesthesia, trial), breathing exercises with an Able Triflow Breathing Exerciser ([Triflow], Able Medical Aids, Largo, FL, USA). - Planned discharge on the fourth postoperative day | |
| Preoperative Evening | - enema for bowel movement regulation | - enema for bowel movement regulation - metabolic priming with 100ml omegaven 10% (Fresenius Kabi AG, Bad Homburg, Germany) - hemodynamic priming with 500ml HES 6% 130/0.42 (Voluven, Fresenius Kabi AG, Bad Homburg, Germany) |
| Two hours before induction of anesthesia | - none | - administration of a high caloric energy drink (ProvideXtra® Drink 200ml, Fresenius Kabi AG, Bad Homburg, Germany) - start of Omegaven 10% continuous infusion; 0,2g/kg/d for 48 hours |
| Anesthesia | - thoracic epidural anesthesia (TEA) - induction with propofol and sufentanil (dosage according to discretion of the physician) - free choice of neuromuscular blocking agent - continuous muscle relaxation during procedure - maintenance with desflurane or sevoflurane at the discretion of the physician, TEA and additional sufentanil, if necessary - TEA bolus of 10ml 0.3% ropivacaine (Naropin, AstraZeneca GmbH, Wedel, Germany) in addition with 10μg sufentanil, - controlled mechanical ventilation on discretion of the physician - insertion of arterial line, central venous line (not trial specific) - extubation directly after end of surgery | |
|  | - 500ml of crystalloid fluid (E153, Serumwerk Bernburg, Bernburg, Germany) for priming of TEA | - 500ml HES 6% 130/0.42 (Voluven, Fresenius Kabi AG, Bad Homburg, Germany) for priming of TEA |
| Surgery | - single shot 1.5 g cefuroxime, 0.5 g metronidazole - liver surgery:   - transverse upper abdomen laparotomy   - insertion of one robinson (drainage) - colon surgery:   - median laparotomy or laparoscopic approach   - no insertion of drainage - intraoperative fluid limited to 4ml/kg/h - blood sugar level <8mmol/L | |
| Postoperative day 0 | - colon surgery: transfer to post anesthesia care unit and then to normal ward - liver surgery: transfer to intensive care unit (ICU) - start of patient controlled TEA (parent solution: ropivacaine 0.2%+ 0.625 mcg/ml sufentanil; 5ml bolus with 20mins time-out; continuous rate 5ml/h; 4hr limit 60ml) - no systemic opioids as along as TEA is working sufficient - infusion limited to 500 ml/day, if patient is drinking appropriately - mobilization 5hrs after end of anesthesia (assisted walking in the room, 2hrs sitting in therapy-chair) - start oral fluids 2 hrs after end of anesthesia (see below) | |
|  | - tea 200 ml, optional yoghurt (maximum fluid 1500 ml) - blood sugar level between 4 mmol/L and 10 mmol/L | - 200ml high caloric energy drink (ProvideXtra® Drink), optional yoghurt (maximum fluid 1500ml) - Omegaven® 10% 0.2g/kg/d - blood sugar level between 4 mmol/L and 8 mmol/L |
| Postoperative day 1 | - patient controlled TEA continuously - no systemic opioids as along as TEA is working sufficient - light fare, oral fluid > 1500 ml - if oral fluid < 1500 ml, iv-fluid (crystalloid E153) possible - mobilization goal: 8 hrs outside of the bed, minimum 2 times assisted walking up and down on the ward floor - breathing exercises (Triflow) - in case of liver surgery shortening of the robinson drainage | |
|  | - blood sugar level between 4 mmol/L and 10 mmol/L | - Omegaven® 10% 0.2g/kg/d - blood sugar level between 4 mmol/L and 8 mmol/L |
| Postoperative day 2 | - removal of TEA catheter, central venous line and robinson drainage (only liver surgery) - if necessary paracetamol per os and patient controlled intravenous opioid analgesia with piritramide (3-5mg bolus, 15-20min time-out) - light fare, oral fluid > 1500 ml - full mobilization goal: >8 hrs outside of the bed, unassisted walking - breathing exercises (Triflow) | |
|  | - blood sugar level between 4 mmol/L and 10 mmol/L | - Omegaven® 10% 0.2g/kg/d - blood sugar level between 4 mmol/L and 8 mmol/L |
| Postoperative day 3 | - if necessary paracetamol per os and patient controlled intravenous opioid analgesia with piritramide (3-5mg bolus, 15-20min time-out) - gradual return to full solid food - oral fluid > 1500 ml - full mobilization goal: >8 hrs outside of the bed, unassisted walking - breathing exercises (Triflow) - Pre-discharge round with patient and relatives, information regarding post hospital course, nutrition counseling | |
|  | - blood sugar level between 4 mmol/L and 10 mmol/L | - blood sugar level between 4 mmol/L and 8 mmol/L |
| Postoperative day 4 | - no systemic opioids, if necessary paracetamol per os - full solid food - oral fluid > 1500 ml - full mobilization goal - discharge on afternoon | |
| Postoperative day 8 | - outpatient appointment, talk about the pathological findings, organization of adjuvant therapy, if necessary - extraction of suture material on 12^th^ postoperative day | |

ERAS: enhanced recovery after surgery, ICU: intensive care unit, MOFA: metabolic optimized fast track concept. TEA: thoracic epidural anesthesia

**Table S3: Blood glucose management**

During the first three postoperative days the blood glucose level was kept between 4 mmol/L and 8 mmol/L in the MOFA or between 4 mmol/L and 10 mmol/L in the control group. Capillary blood sugar level was measured four times daily. If the blood sugar level exceeded 8 mmol/L short acting alt-insulin was administered subcutaneously according to the following table:

| **Blood sugar level [mmol/L]** | **Alt-insulin [I.U.]** |
| --- | --- |
| 8.0-8.9 | 4 |
| 9.9-10.9 | 5 |
| 11.0-12.9 | 6 |
| 13.0-13.9 | 7 |
| >14.0 | Call physician |

I.U.: international units

**Additional Results**

**Figure S1 Blood sugar levels**





A: Blood sugar level before insulin treatment, B: Blood sugar level after insulin treatment. Values are given as mean ± standard deviation. Statistical significance was considered to be at two-sided p<0.05. Differences between groups, as well as time and time vs. group effect were tested using a general linear model with the respective baseline value as covariate. a: linear effect, D0: postoperative evening 8 p.m., 12, 16,…,72 hours after D0, respectively. MOFA: metabolic optimized fast track concept.

**Figure S2 Coagulation**





Values are given as boxplot with whiskers minimum to maximum. Statistical significance was considered to be at two-sided p<0.05. Differences between groups, as well as time and time vs. group effect were tested using a general linear model with the respective baseline value as covariate. a: linear effect, b: quadratic effect, BL: baseline, D1-D3:postoperative day 1 or 3, respectively, Dis: discharge. aPPT: activated partial thromoplastin time, MOFA: metabolic optimized fast track concept.

**Figure S3 Parameters of liver injury and function**





Figure S3: Parameters of liver injury and function. Values are given as boxplot with whiskers minimum to maximum. Statistical significance was considered to be at two-sided p<0.05. Differences between groups were tested at D1, D3 and Dis using a Mann-Whitney *U* test. No statistical significant differences between groups were detected (detail p-values are not shown). ATIII: antithrombin 3, Bilirubin: unconjugated bilirubin, γ-GT: gamma-glutamyltransferase, ASAT: aspartate aminotransferase, ALAT: alanine aminotransferase, CHE: cholin esterase, MOFA: metabolic optimized fast track concept.

**Figure S4 Inflammation**



Values are given as mean ± standard deviation. Statistical significance was considered to be at two-sided p<0.05. Differences between groups, as well as time and time vs. group effect were tested using a general linear model with the respective baseline value as covariate. a: linear effect, b: quadratic effect, BL: baseline, D1-D3:postoperative day 1 or 3, respectively, Dis: discharge. IL6: Interleukin 6, IL-10 Interleukin 10, TNF-α: tumor necrosis factor-α, WBC: white blood cell count, CRP: c-reactive protein, PCT: procalcitonin, MOFA: metabolic optimized fast track concept.

|  |  | **Time Point** | | | | | **P value** | | |
| --- | --- | --- | --- | --- | --- | --- | --- | --- | --- |
| **Variable** |  | **BL** | **EoS** | **D1** | **D2** | **D3** | **Group** | **Time** | **Time*Group** |
| **MAP** | Control [38] | 100 ± 16 | 87 ± 13 | 86 ± 13 | 91 ± 13 | 98 ± 14 | 0.071 | 0.143 | 0.587 |
| [mmHg] | MOFA [42] | 96 ± 11 | 83 ± 10 | 84 ± 11 | 87 ± 12 | 91 ± 11 |  |  |  |
| **HR** | Control [38] | 73 ± 9 | 75 ± 13 | 82 ± 13 | 83 ± 12 | 80 ± 12 | 0.830 | 0.291 | 0.171 |
| [bpm] | MOFA [42] | 75 ± 9 | 80 ± 13 | 81 ± 12 | 83 ± 12 | 78 ± 12 |  |  |  |
| **Temp** | Control [37] | 36.6 ± 0.5 | 36.9 ± 0.9 | 37.4 ± 0.5 | 37.3 ± 0.6 | 37.1 ± 0.6 | 0.932 | 0.183 | 0.992 |
| [°C] | MOFA [42] | 37.4 ± 0.4 | 36.9 ± 0.8 | 37.5 ± 0.6 | 37.3 ± 0.5 | 37.1 ± 0.5 |  |  |  |

Table S4: Hemodynamic data. Values are given as mean ± standard deviation. Number of analyzed patients can be found in brackets. Statistical significance was considered to be at two-sided p<0.05. Differences between groups, as well as time and time vs. group effect were tested using a general linear model with the respective baseline value as covariate. BL: baseline, EoS: End of surgery, D1-D3:postoperative day 1-3, respectively, MAP: mean arterial pressure, HF: heart rate, Temp: temperature, MOFA metabolic optimized fast track concept.

| **Variable** | **Control** | **MOFA** | **P value** |
| --- | --- | --- | --- |
| **Need for Insulin therapy**  All  D0  D1  D2  D3 | 13 (37.1) [35]  11 (31.4) [35]  8 (25.0) [32]  5 (15.6) [32]  3 (9.4) [32] | 32 (69.6) [46]  24 (53.3) [45]  18 (40.0) [45]  13 (29.6) [44]  9 (21.4) [42] | 0.006  0.070  0.224  0.174  0.212 |
| **Insulin dosage (I.U.)**  Cumulative  D0  D1  D2  D3 | 10.0 (2.0, 4.0, 30.0, 102.0) [13]  5.0 (4.0, 4.0, 10.0, 12.0) [11]  10.0 (4.0, 4.0, 43.0, 72.0) [8]  10.0 (4.0, 4.5, 10.5, 11.0) [5]  6.0 (5.0, 5.0, 7.0, 7.0) [3] | 9.0 (4.0, 4.3, 17.5, 57.0) [32]  5.0 (4.0, 4.3, 9.5, 14.0) [24]  7.0 (4.0, 4.0, 10.5, 22.0) [18]  6.0 (2.0, 5.0, 9.5, 15.0) [13]  5.0 (4.0, 4.5, 17.5, 25.0) [9] | 0.906  0.649  0.276  0.673  n.a. |

Table S5: Insulin therapy. Values are given as median (minimum, 25% percentile, 75% percentile, maximum) or absolute number (percentage) as appropriate. Number of analyzed patients can be found in brackets. Statistical significance was considered to be at two-sided p<0.05. Differences between groups were tested using a Student *t*-test or Mann-Whitney *U* test as appropriate. Frequencies were analyzed using Chi-square or Fisher’s Exact test, as appropriate.D0-3: day of surgery and postoperative day 1-3, respectively, I.U.: international unit, MOFA: metabolic optimized fast track concept, n.a.: not applicable.

|  |  | **Time Point** | | | | | | **P value** | | |
| --- | --- | --- | --- | --- | --- | --- | --- | --- | --- | --- |
| **Variable** |  | **BL** | **EoS** | **D1** | **D2** | **D3** | **Dis** | **Group** | **Time** | **Time*Group** |
| **Na** | Control [36] | 141.3 ± 2.3 | 141.5 ± 2.3 | 139.8 ± 3.0 | 139.9 ± 2.3 | 140.6 ± 2.6 | 140.8 ± 3.1 | 0.185 | 0.052 | 0.189 |
| **[mmol/L]** | MOFA [42] | 140.5 ± 2.6 | 141.6 ± 3.6 | 138.9 ± 2.9 | 138.6 ± 3.4 | 138.9 ± 3.4 | 139.9 ± 3.0 |  |  |  |
| **K** | Control [36] | 4.2 ± 0.4 | 4.2 ± 0.4 | 4.0 ± 0.4 | 3.9 ± 0.3 | 3.9 ± 0.4 | 4.1 ± 0.4 | 0.850 | 0.028b | 0.010a |
| **[mmol/L]** | MOFA [42] | 4.2 ± 0.5 | 4.1 ± 0.3 | 4.0 ± 0.5 | 3.9 ± 0.4 | 3.9 ± 0.4 | 4.3 ± 0.5 |  |  |  |
| **Creatinine** | Control [36] | 82 ± 19 | 76 ± 21 | 83 ± 30 | 82 ± 52 | 76 ± 45 | 76 ± 28 | 0.883 | 0.001b | 0.757 |
| **[μmol/L]** | MOFA [42] | 72 ± 11 | 68 ± 14 | 73 ± 17 | 65 ± 13 | 66 ± 13 | 69 ± 12 |  |  |  |
| **BUN** | Control [36] | 5.4 ± 1.3 | 5.1 ± 1.5 | 6.3 ± 2.4 | 5.9 ± 3.4 | 5.4 ± 3.6 | 5.1 ± 3.9 | 0.244 | 0.830 | 0.890 |
| **[mmol/L]** | MOFA [42] | 5.2 ± 1.6 | 4.5 ± 1.3 | 5.9 ± 1.7 | 5.0 ± 1.7 | 4.7 ± 2.0 | 4.5 ± 1.5 |  |  |  |
| **Hb** | Control [36] | 8.2 ± 1.3 | 7.3 ± 1.2 | 6.9 ± 1.0 | 6.7 ± 1.1 | 6.8 ± 1.2 | 7.3 ± 1.0 | 0.262 | 0.108 | 0.405 |
| **[mmol/L]** | MOFA [38] | 8.4 ± 0.8 | 7.3 ± 1.1 | 6.9 ± 1.0 | 6.7 ± 0.9 | 6.8 ± 0.9 | 7.1 ± 1.0 |  |  |  |
| **Hct** | Control [36] | 0.38 ± 0.05 | 0.35 ± 0.05 | 0.33 ± 0.04 | 0.32 ± 0.05 | 0.33 ± 0.05 | 0.35 ± 0.04 | 0.303 | 0.022a | 0.187 |
| **[%]** | MOFA [38] | 0.39 ± 0.03 | 0.35 ± 0.05 | 0.33 ± 0.05 | 0.32 ± 0.04 | 0.32 ± 0.04 | 0.34 ± 0.04 |  |  |  |
| **MCV** | Control [36] | 86.1 ± 6.0 | 86.0 ± 6.5 | 86.1 ± 6.3 | 87.0 ± 5.9 | 86.2 ± 5.8 | 86.4 ± 5.7 | 0.825 | 0.060 | 0.154 |
| **[fL]** | MOFA [38] | 88.7 ± 4.4 | 88.6 ± 3.5 | 88.7 ± 4.2 | 88.7 ± 4.1 | 88.8 ± 3.8 | 88.8 ± 3.8 |  |  |  |
| **PLT** | Control [36] | 260 ± 111 | 219 ± 115 | 210 ± 95 | 197 ± 85 | 208 ± 80 | 317 ± 107 | 0.578 | <0.001b | 0.245 |
| **[Gpt/L]** | MOFA [38] | 274 ± 92 | 225 ± 88 | 220 ± 82 | 210 ± 89 | 230 ± 95 | 351 ± 108 |  |  |  |

Table S6: Additional clinical chemistry and blood count. Values are given as mean ± standard deviation. Number of analyzed patients can be found in brackets. Statistical significance was considered to be at two-sided p<0.05. Differences between groups, as well as time and time vs. group effect were tested using a general linear model with the respective baseline value as covariate. a: linear effect, b: quadratic effect, BL: baseline, EoS: End of surgery, D1-D3:postoperative day 1-3, respectively, Dis: discharge. Na: serum sodium level, K: serum potassium level, BUN: blood urea nitrogen, Hb: hemoglobin, Hct: hematocrit, MCV: mean corpuscular volume, PLT: platelets.
